# Exploring the Relationship Between Changes in Weight and Utterances in an Online Weight Loss Forum: A Content and Correlational Analysis Study

**DOI:** 10.2196/jmir.3735

**Published:** 2014-12-08

**Authors:** Eric B Hekler, Gaurav Dubey, David W McDonald, Erika S Poole, Victor Li, Elizabeth Eikey

**Affiliations:** ^1^School of Nutrition and Health PromotionArizona State UniversityPhoenix, AZUnited States; ^2^Pennsylvania State UniversityUniversity Park, PAUnited States; ^3^University of WashingtonSeattle, WAUnited States

**Keywords:** social media, social support, weight loss, Natural Language Processing

## Abstract

**Background:**

There is increasing interest in the use of online forums as a component of eHealth weight loss interventions. Although the research is mixed on the utility of online forums in general, results suggest that there is promise to this, particularly if the systems can be designed well to support healthful interactions that foster weight loss and continued engagement.

**Objective:**

The purpose of this study was to examine the relationship between the styles of utterances individuals make on an online weight loss forum and week-to-week fluctuations in weight. This analysis was conducted to generate hypotheses on possible strategies that could be used to improve the overall design of online support groups to facilitate more healthful interactions.

**Methods:**

A convenience sample of individuals using an online weight loss forum (N=4132) included data both on online forum use and weight check-in data. All interactions were coded utilizing the Linguistic Inquiry and Word Count (LIWC) system. Mixed model analyses were conducted to examine the relationship between these LIWC variables and weight over time.

**Results:**

Results suggested that increased use of past-tense verbs (*P*=.05) and motion (*P*=.02) were associated with lower weekly weights whereas increased use of conjunctions (eg, and, but, whereas; *P*=.001) and exclusion words (eg, but, without, exclude; *P*=.07) were both associated with higher weight during the weeks when these utterances were used more.

**Conclusions:**

These results provide some insights on the styles of interactions that appear to be associated with weight fluctuations. Future work should explore the stability of these findings and also explore possibilities for fostering these types of interactions more explicitly within online weight loss forums.

## Introduction

### Background

The Internet is becoming a place for health information and social support. A recent Pew Research Center survey reported 72% of respondents search for health-related information online at least once a year, with more than a quarter of survey respondents specifically seeking experiences of others with similar health concerns [1]. Online communities for health not only offer social support [2-4], accountability [5], and peer modeling [6], but can also extend the reach of care for patients who fall between the cracks of traditional health care. Based on this, there is great potential for online forums as a key part of systems that foster health behavior change.

Although there is great promise, current evidence supporting the utility of online communities is limited [7-9]. Specifically, these online communities, including social media and online forums/message boards, often promote only modest behavior change. This seems to be particularly the case because of high attrition rates. Based on this, there is great potential for these systems to promote behavior change and weight loss at scale, but additional research is required to better understand how best to design the systems to facilitate continued use and weight loss.

One strategy that could be utilized to improve the design of these systems is to explore how interactions within a system relate to outcomes such as weight. A great deal of research from psychology has articulated styles of interactions during therapeutic sessions that are predictive of behavior change. For example, empathic regard by a therapist and committal statements made by a client, particularly at the end of the session, are both predictive of improved clinical outcomes, such as reduced alcohol consumption [10-12]. Beyond therapeutic style, previous research by Pennebaker and colleagues [13,14] have provided a solid foundation for computationally documenting and understanding interaction styles from written documents (more subsequently). Collectively, this work suggests that there is likely great potential for understanding how best to improve online forums by articulating the interactions that appear to be associated with improved outcomes.

This paper reports findings concerning the relationship between styles of utterances in an online forum and weight loss via data provided to us from the makers of DropPounds (note: name changed to protect confidentiality). DropPounds is one of the most popular weight loss smartphone and Web-based online weight loss forums currently on the market. Users of DropPounds can specify various types of personal goals (eg, target weight, exercise, measurements, nutrition). DropPounds then prepares personalized targets to strive for and, in the paid premium plans, detailed plans. DropPounds provides a companion application for smartphones, tablets, and PCs. This application allows users to log their exercise and calorie consumption. The service also integrates with wireless activity and weight tracking devices, including the Withings scale, Nike+, and Fitbit. DropPounds provides peer support in the form of an online forum, along with social networking features such as private messaging and friend lists. The forum can be used to facilitate team and open challenges. It also provides a venue for discussing health, nutrition, exercise, personal reflection and experiences, and various other topics (eg, see [15]). In this paper, we examine how different utterances of participants in the DropPounds online forums, as codified by a validated natural language processing tool called Linguistic Inquiry and Word Count (LIWC), predict week-to-week fluctuations in weight among DropPounds forum users.

### Related Research

#### Internet-Assisted Weight Loss Interventions

Although online forums are used for many purposes, research studying the effect of online forums as a tool to support weight loss is limited. The vast majority of research on online weight loss interventions (eg, Tate et al [16], Womble et al [17], Finley et al [18], Harvey-Berino et al [19], Neve et al [20,21], and Weinstein [22]) examines the effect of Internet-assisted weight loss intervention packages that included an online forum as 1 of its components. There is far less work examining characteristics of the forum itself that might predict health outcomes. Bensley et al [23] conducted one such study; they reviewed online groups and generated 5 possible design suggestions, including (1) recreate the human experience, (2) personalize, (3) create a dynamic experience, (4) provide a supportive environment, and (5) build on sound theory. This research was largely qualitative, however, with limited empirical data supporting these design suggestions. There is clearly potential for deeper research in this area. Our research attempts to fill this research gap by examining the effects of forum activity on weight loss.

#### Language Inquiry and Word Count

LIWC is a linguistic analysis tool [13,24-26]. It consists of a dictionary mapping words and word stems to linguistic categories, as well as a software program that calculates various metrics on text data using this dictionary. (Note that we did not use the program itself for this project. Because the amount of data we were working with was beyond the tool’s abilities, we used a custom version of a Perl script.) The purpose of LIWC is “to provide a method for studying psychological phenomena (eg, emotion, cognition) that are present in individuals’ communications” [24,25].

When LIWC processes a piece of text, counts are maintained corresponding to each category in its dictionary. When a word is scanned, the counts for all dictionary categories that include that word are incremented. The output consists of the values for all counts once the entire text has been scanned.

#### Dictionary

LIWC’s dictionary assigns words and word stems to various categories (eg, positive emotion, anxiety, pronouns). The categories of the current dictionary can themselves be categorized in the following ways [13,24-26]: (1) 22 standard linguistic dimensions (eg, pronouns, articles, auxiliary verbs), (2) 32 categories tapping psychological constructs (eg, affect, cognition, biological processes), (3) 7 personal concern categories (eg, work, home, leisure activities), and (4) 3 paralinguistic dimensions (assents, fillers, nonfluencies).

LIWC’s dictionary has gone through multiple iterations of compilation and revision [13]. Initially, there were only 2 categories—positive and negative emotion—but this was expanded during the original round of compilation as the compilers added more types of language they wanted to capture. Initial word lists were assembled from various sources (eg, dictionaries) and assessed by groups of independent judges. Initial compilation was done between 1992 and 1994, and revisions were done in 1997 and 2007 [13].

#### Linguistic Inquiry and Word Count Validation

LIWC has been validated through a variety of mechanisms. For example, in a writing study, college students were asked to write essays from both emotional and nonemotional standpoints. These essays were rated using both LIWC and a panel of 4 independent judges [27]. Later analyses of these data found that the LIWC scales and judges’ ratings were highly correlated [13,24-26].

There is a research precedent for using LIWC analysis of text to study various behavioral and psychological issues. For instance, Rude et al [28], Gunsch et al [29], and Kowalski [30] used LIWC analysis to gain insight into the cognitive operations associated with depression, the affective composition of political campaign ads, and the emotional perception of teasing, respectively.

On the other hand, the use of LIWC metrics as predictive tools has also been investigated. For instance, Simmons et al [31], Sexton and Helmreich [32], and Leshed et al [33] used LIWC metrics to predict marital health, pilot performance, and performance during collaborative tasks. We have applied this research methodology to the current task. DropPounds is ideal for this sort of analysis because natural writing is a fundamental part of its feature set and the sort of disclosures that occur within the forums map on to the intended purpose of LIWC focused on self-disclosure [27].

### Research Questions

#### Overview

We explored 4 different models examining the relationship between utterance styles and weekly fluctuations in weight. Note that for each model we also included word count in all models as a control for overall verbal fluency.

#### Model 1

The first model focused on discussions about health. It is logical to presume that individuals who tend to talk more about health, consumption, or general biological mechanisms of weight loss may be more actively engaging in their weight loss during those times and, thus, lose more weight. Based on this presumption, we focused on content words as our first model.

#### Model 2

The second model focused on emotion. Specifically, it is plausible that content words related to positive and negative affect would be predictive of weight fluctuations with more positive affect associated with weight loss and more negative affect associated with weight gain.

#### Model 3

In our third model, we chose groupings of words that have been linked to psychological phenomena in previous research that could impact weight loss, including (1) social process words (eg, talk, they, child) which have been linked to social support; (2) past tense use (eg, went, ran, had), which suggests a focus on the past; (3) certainty (eg, always, never), which has been linked with emotional stability; (4) exclusive words (eg, but, without, exclude), which have been linked with honesty; and (5) third-person singular (eg, she, her, him), which has been linked to exhibiting social support [13,14].

#### Model 4

The fourth model was data-driven, in that we chose styles of utterances that were observed in our dataset most often within the forums and could be indicative of important areas to focus on within online weight loss forums. This fourth model specifically included past tense use, conjunctions, and “motion” words (eg, arrive, go, car).

Per Pennebaker et al [13,14], although a lot of initial emphasis was placed on “content” words (eg, emotions and personal concerns) in early development of LIWC, research increasingly indicated that function or “style words” (eg, pronouns, prepositions, verb tense) often had higher association with phenomena under study. As such, we anticipated the majority of findings to be localized around these style words, but we did develop content-related explorations based on their intuitive appeal and because there is limited research exploring these questions.

## Methods

### Data Preparation

Two datasets provided by the makers of DropPounds were combined to conduct these analyses. The first included the following data at a weekly level for users of DropPounds: weight, number of active days per week, calorie intake budget, number of calories consumed, and number of calories burned through exercise. In addition to users entering data manually, a subset of users also used Withings brand wireless-enabled weight scales. Although users may check in these data several times a week, when we received the data, it had been aggregated and reported on a weekly basis. Every check-in’s weight was measured at the end of a weekly interval. This interval always started on the same day of the week—either Sunday or Monday, depending on the user. This check-in date was created based on the data provided to us (ie, the weekly weight check-in data was always aggregated on a Sunday or Monday). Identifying each user’s check-in day was necessary for aggregating weekly LIWC metrics from the values for individual posts.

The second dataset included all forum posts of the participants. Because weight and other health data check-ins were aggregated weekly, we also aggregated forum-derived data at the weekly level using the same start weekday. For each post, we generated LIWC metrics.

After data cleaning, the dataset included weekly weight loss progress and forum check-ins from DropPounds users between July 2008 and October 2012. We processed the forum posts using the LIWC linguistic analysis tool, generating content metrics for each forum post based on a dictionary that assigns words of the English language to various topic categories as already discussed. These metrics were used as predictors in a multilevel mixed model analysis, with user weight as the predicted outcome variable.

Participants within the study were self-selected. Specifically, the act of joining the forum occurred via finding the forum online (either via being directed from DropPounds mobile app or likely via a Google Search). There was no other active recruitment for this forum beyond standard commercial practices that might be deployed by a commercial weight loss company.

#### Linguistic Inquiry and Word Count Metrics

LIWC results were generated for every single forum post in the forum dataset and then aggregated to the weekly level to conform to the weight metric. Because LIWC metrics are word counts, aggregating each LIWC metric for a given user over a given week simply involved adding up the values of the metric for the user’s posts over that week. Word count was controlled for in all models.

### Data Cleaning

#### Data Anomalies

The weekly check-in data contained a number of anomalies, such as outliers, out-of-range check-ins (ie, automatically generated check-ins from before the earliest or after the latest possible check-in date), and sequences of check-ins that consisted of the same weight carried forward from previous weeks. We applied the following filters and corrections to clean the dataset.

1. If 2 or more consecutive check-ins had diet and exercise check-in values of zero (an indicator of nonreporting) and they had identical weight, then we assumed that those consecutive weight check-ins were automatically generated and filtered them out of our dataset.
2. If a check-in had nonzero diet and exercise check-in data, and was followed by a check-in with identical weight and diet and exercise check-in data that equaled zero, we assumed the second check-in was automatically generated by copying the first and we filtered out the second check-in.
3. We flagged all users with weight check-ins over 600 lb for outlier investigation.
4. We examined the flagged data and corrected anomalous weight entries where possible, based on surrounding entries (ie, a string of entries with weight 179 lb contained a single check-in with weight 1111 lb and this was corrected by inspection).
5. We used linear interpolation to correct sequences of repeated outlier check-ins.
6. We deleted all users who showed no change in weight throughout their data-gathering period.
7. Two users had parallel data streams. We deleted the automatically generated streams, leaving the manually checked-in data.
8. We calculated the change in weight from 1 week to the next for each check-in. If there was a large weight change between check-ins, we took the length of time gap was taken into account before applying outlier processing. To filter out users with unreasonable changes in weight, we only kept check-ins for users who had a maximum absolute weekly weight change of 5 pounds or less, which is conceptually similar to strategies used by Finley et al [18].

In applying these changes, we brought the size of the dataset from more than 1 million check-ins to approximately 100,000, representing approximately 4000 users. However, further data preparation was necessary before we could begin modeling. We describe this process in the following section.

To provide some context for our statistical results, we included text from the forums in the Post Text Results section within the current paper. Note that slight modifications to the posts were made if any potentially identifying information was provided in the text. To find these posts, we selected the top 10 posts for each category (ie, high in the particular style word use, such as past-tense verbs). We then selected passages from this group that were the best demonstration of the general style of conversation found among those 10 posts.

### Statistical Analyses

Similar to Neve et al [20], we chose multilevel mixed models as our analysis technique [34]. The advantage of this technique is that it is capable of dealing with observations that are nested (eg, multiple observations for each individual), have unbalanced data (where individuals have unequal observations), and contain missing values.

Prior to conducting any mixed model analyses, variables are recentered to make the results more readily interpretable. A recentered variable is restated as a deviation from a theoretically meaningful reference value (eg, time since first observation instead of calendar date and time).

In our dataset, we recentered the starting date for each week. This was done by representing starting dates as week numbers. Each starting date was replaced by the number of weeks between the starting date and the date when the user first used DropPounds.

Multilevel mixed models were developed using an iterative methodology. Each model attempted to improve on the previous model, reducing the residual error and explaining more of the target variance. Here, we describe the models generated.

The first model generated was an unconditional means model. This model included no predictors and attempted to express the target variable in terms of a grand mean (the fixed intercept) and a user-specific deviation from the grand mean (the random intercept). This model served as a baseline for model comparison. If the random effect was not statistically significant, we concluded that the individual-level mean target values were not statistically different than the grand mean target value for the entire dataset.

The next set of models added time as a predictor. These were the unconditional growth models and they attempted to determine the effect of time on the target variable. Multiple unconditional growth models were developed. Each included a higher-order power of time than the previous. This enabled us to study curvilinear change over time, if there was a plausible reason to anticipate such change (as was the case with weight whereby it was plausible for a quartic linear effect based on the well-studied concept of “yo-yo” dieting with weight loss, plateaus, and then weight gain again and another plateau). Finally, we generated the models that include the predictors of interest after controlling for time and for overall word count.

## Results

### Mixed Model Results

Table 1 reports descriptive statistics about the sample (N=4132). In brief, the mean age was 37.5 (SD 12.4) years and most were women (81.60%, 3371/4132)*.* These values are similar to those seen in other studies focused on commercial weight loss interventions [18,20].

**Table 1:** Descriptive statistics of the sample (N=4132).^a^

Variables	Sample	Range
Age (years), mean (SD)	37.5 (12.4)	18-87
Starting weight (lbs), mean (SD)	190.1 (46.7)	63-483
Number of weeks actively checked-in weight and used forum, mean (SD)	23.5 (23.6)	2-185
Women, n (%)	3371 (81.60)	

^a^ Because these data were provided to us, “normal” demographic data for scientific publications (eg, ethnicity, BMI) were not available.

Results comparing the unconditional means model to the unconditional growth models indicated that there was a significant impact of time on weight. Specifically, results indicated that there was a significant effect of linear and quadratic time. These results suggested that individuals utilizing the forum on DropPounds tended to lose weight and then their weight tended to level off. Results exploring if there was a cubic (which could be indicative of weight regain) or quartic (which could be indicative of yo-yo weight loss of regain, loss, and regain) revealed that there was not sufficient variance within the model at later stages to properly test for cubic or quartic effects. Based on this, the unconditional growth model that included a random linear time predictor and a fixed linear and quadratic predictor was used (ie, common metrics among all models in Table 2, excluding word count). In the model whereby only word count was added to the model, results found that word count alone did not predict weight (ie, *B*=–0.000009, SE 0.000063, *P*=.13). That said, results indicated a significant model fit improvement when word count was added compared to the unconditional growth model that included quadratic time (ie, unconditional growth model: –2 log-likelihood [–2LL]=621186, Akaike information criterion [AIC]=621198, Bayesian information criterion [BIC]=621240; word count model: –2LL=588920, AIC=588934, BIC=588984). Based on this improved model fit and based on the need to control for overall usage, all LIWC-specific predictor models were compared to the unconditional growth model and the word count model for determination of improved model fit (see Table 2).

**Table 2:** Mixed models, model fit statistics.

Predictor variables	Unconditional growth	Word count model	Model 1	Model 2	Model 3	Model 4
-2 Log-likelihood	621186	588920	588915	588919	588909	588898
AIC	621198	588934	588935	588937	588933	588919

The word count model represents the unconditional growth model, which includes a random linear effect and a fixed linear and quadratic effect of time, as well as word count per week per participant. Model 1 represents analyses that included topic-relevant words, such as health, ingestion, and motion. Model 2 represent analyses that include positive and negative affect words. Model 3 represents theoretically meaningful words including social process, past-tense verb use, certainty, exclusion, and third-person singular words. Model 4 represents analyses that include conjunction, motion, and past-tense verb use. With regard to –2LL and AIC, lower values indicate better model fit.

Table 3 reports results of the 4 models. In general, results suggest that the content words explored in model 1 (ie, health, ingestion, and biology) and model 2 (ie, positive and negative emotion words) were not predictive of fluctuations in weight from 1 week to the next. Results from the theoretically driven model (model 3) indicated that use of past-tense verbs was predictive of weight loss. Specifically, when individuals utilized more past-tense verbs in their writing (an indication of thinking about the past), they tended to have lower weight that week relative to other weeks. In addition, use of exclusion words was also predictive of fluctuations in weight. Specifically, during weeks when individuals utilized more exclusion words, which in previous work has been linked to increased honesty, there was a statistical trend for those individuals to exhibit higher weight during the same week. The final model, which was more data-driven, found that the 3 LIWC variables with the most variance were all associated with weekly fluctuations in weight. In particular, increased use of past-tense verbs and discussions about motion, which is a content word that could be indicative of physical activity, during the week were associated with lower weights during those weeks. Finally, results indicated that increased use of conjunctions, which has not been linked with any psychological variables in the past, was associated with increased weight during those weeks. Examination of the model fit statistics (ie, –2LL, AIC, and BIC) indicated that model 4 exhibited the greatest overall model fit.

**Table 3:** Mixed models predicting weekly changes in weight.

Predictor variables	Model 1		Model 2		Model 3		Model 4	
	B (SE)	*P*	B (SE)	*P*	B (SE)	*P*	B (SE)	*P*
Fixed effects								
Fixed intercept	187.1 (0.5011)	<.001	187.1 (0.5011)	<.001	187.1 (0.5011)	<.001	187.1 (0.5011)	<.001
Time	–0.2817 (0.0059)	<.001	–0.2817 (0.0059)	<.001	–0.2817 (0.0059)	<.001	–0.2816 (0.0059)	<.001
Quadratic time	0.0016 (0.0000)	<.001	0.0016 (0.00002)	<.001	0.0016 (0.0000)	<.001	0.0016 (0.0000)	<.001
Word count	–0.0004 (0.0002)	.03	–0.0002 (0.0003)	.62	–0.0001 (0.0004)	.76	–0.0008 (0.0004)	.07
Model 1								
Health	–0.0153 (0.0139)	.27						
Ingestion	–0.0002 (0.0096)	.98						
Biology	0.0112 (0.0106)	.29						
Model 2								
Positive emotion			0.0069 (0.0083)	.41				
Negative emotion			–0.0068 (0.0097)	.48				
Model 3								
Social process					–0.0028 (0.0034)	.40		
Past-tense verbs					–0.0164 (0.0061)	.007		
Certainty					–0.0126 (0.0157)	.42		
Exclusion					0.0148 (0.0082)	.07		
Third-person sing					0.0202 (0.0134)	.13		
Model 4								
Conjunction							0.0203 (0.0063)	.001
Motion							–0.0191 (0.0084)	.02
Past tense							–0.0109 (0.0056)	.05
Random effects								
Linear time	0.121 (0.003)	<.001	0.121 (0.003)	<.001	0.121 (0.003)	<.001	0.121 (0.003)	<.001
Random intercept	1994.6 (31.7)	<.001	1994.5 (31.7)	<.001	1994.5 (31.7)	<.001	1994.5 (31.7)	<.001
Residual	16.4 (0.082)	<.001	16.4 (0.082)	<.001	16.4 (0.082)	<.001	16.4 (0.082)	<.001

### Post Text Results

#### Overview

To give some context to results of the mixed model analyses, some examples of posts that exemplify the different types of utterance usages are provided subsequently.

#### Past-Tense Verbs

Posts with past-tense verbs use, which was associated with lower weight during weeks when they were used, tended to compare a person’s current weight situation to the past. Often, these posts were very long and some even started from childhood and worked forward from there as a sort of “weight loss journey” chronicle. For example, here are excerpts from 1 forum user:

##### Past Tense Example 1

I have struggled with my weight since third grade. I have tried so many different diets—a childhood friend and I were first introduced to WW when we were in the fifth grade, needless to say it really didn’t work...This was the first time in years that I was looking thinner, feeling better about myself and drastically dropping dress sizes—clothes shopping became fun! I got down to 170-175, yeah, I know that still sounds heavy, but I carry my weight differently and looked 20 pounds lighter than I actually was...After graduating, my husband and I thought that our life was starting, we would start our family and everything would just be right. In 2005 we unexpectedly became pregnant...my heaviest weight is 339.6 lb and I am only 5′4″, my goal weight seems so far away at 185 lb. So far, this program has been working well—it is easy to follow, now I just need to exercise.

##### Past Tense Example 2

I feel you—I think the biggest part of me being apprehensive to this whole weight loss thing again (in terms of food) was counting calories. 1) I was terrified of finding out what I consume, considering I work out pretty meticulously and wasn’t losing a thing, and 2) I was daunted at how much work it takes. Eating out becomes a headache, I was scared of having to measure everything, what happens when I just don’t KNOW the calories-do I avoid it all together? I fell off the “healthy lifestyle” wagon after few illnesses that happened right after the other. I couldn’t exercise and started eating whatever I wanted. But realistically, it does become second nature. I lost 110 the last time I “counted” and only actively counted. Eventually it not only became habit (knowing WHAT to eat and how much) but I started becoming full off of what I NEEDED vs wanted. My dad just knew how many points everything was and knew how to subconsciously eye out portions. A big thing, which I think not too many people think of, is our cravings and their relation to our taste buds and what our body is taking in nutritionally...You can literally train your body into WANTING healthier foods...So, I think, not only do watching calories and what you eat become second nature, but it becomes fulfilling and preferable.

#### Motion-Related

With regard to the motion-related posts, a scan of the posts with the highest use of motion words tended to revolve around 2 general themes: most commonly a detailed report about an exercise routine, race/run/marathon, and far less often the high motion posts were detailed travel reports on a recent life-changing vacation/trip that inspired weight loss in some way. For example, here are some short excerpts from 1 of the posts reporting on an exercise routine/race:

##### Motion Exercise Activity Example 1

Yesterday I ran (maybe run is too strong) the St. Jude Memphis Marathon. I’ve been training for months. If you count the time building up a solid running base, training has actually been almost 2 years. After my first half in April, I felt good afterwards and figured I had it in me to try the full distance. I wanted the feeling of not being sure I could finish the race. The half marathon never made me feel like that. I was told the marathon would deliver. I was told correctly.

##### Motion Exercise Activity Example 2

What is the most common thing you see women do in the gym? Cardio. And if they do lift weights they pick up a 5-pound dumbbell and do endless reps. As we have discussed, women need to lift heavy, challenging weights just like men in order to gain muscle. While machines do provide sufficient stimulation to gain muscle, nothing can beat free-weight/compound exercises. Now, we will go over a few free-weight and compound exercises that we will incorporate in the weight training presented.

Followed by definitions of each exercise and then a workout schedule

And here is an example of the beginning of the tale of a “life-changing” trip:

##### Motion Trip Example 1

So this trip I just took. It was a trip of a lifetime. It ranks up there with my wedding, my brother’s wedding in Scotland, and in a way it might be even more significant than even I realize right now. Something happened while on this trip. I turned a corner in this journey. I said goodbye to that girl, the one who has been with me my entire life. I let go of my inner fat girl. I love her, but I don’t need her anymore...I felt something inside of me click into place, I felt like I had ARRIVED...

#### Exclusion-Related

When examining the posts that tended to be related to exclusions (and were associated with slight weight gain), the posts often suggested that the person was providing explanations for a failed plan at weight loss. For example, here are 2 from forum users:

##### Exclusion Example 1

I lost almost a year to a serious foot injury that was misdiagnosed and then turned out to be an injury that heals slowly. I had been exercising regularly up until then and had lost some weight, but gained it all back plus 15 pounds more during my enforced inactivity. I’ve just started exercising regularly and have just added [DropPounds] to the equation. I’m not an enthusiastic exerciser either, but I have given myself a few options and I can change it up enough to stick with it. If the gym doesn’t work, see what you can do at home. My cable provider has Exercise on Demand; I haven’t tried them because that’s not really my thing, but I did notice them. If you have cable, you might want to check out what’s available. You don’t need a lot of room or fancy equipment. Forget the gym, because if it’s that much hassle it’s easy to talk yourself out of it. If you don’t make it easy for yourself to do, you won’t do it.

##### Exclusion Example 2

Here is an example that happened to me recently. I went home to visit my mom for a long weekend, and ate out a lot. I ate in a reasonable manner, only went overbudget 1 day. I also did a 16-mile run with hard, fast segments in it, and ran at least an hour every day. I was 6 pounds heavier when I got home. My fingers were so swollen I could not get my wedding ring off. After about 4 days of eating food I was accustomed to, I was back to my normal weight. I am convinced it was all the sodium. My mother also salts everything as if she needs to preserve her food before eating it. It’s ridiculous...I was once on an 8-week plateau during my weight loss journey. I didn’t change anything up; eventually I worked through it. Sometimes just sticking with the plan works. My husband (who has lost 120 pounds) was more of a “cycler” mixing it up if he hit a plateau. He also does not count calories, but focuses on eating whole foods and being aware of his appetite and eating only when he is hungry.

#### Conjunctions

In a similar vein to exclusion words, use of conjunctions was also associated with modestly higher weight during those weeks. When reading through posts that utilized many conjunctions, responses tended to have a sort of “lessons learned” flavor to them. For example:

##### Conjunction Example 1

I relearned today NOT [sic] to eat cereal! More on that in a moment. If I run right after I get up and am not starving, I won’t eat beforehand. If I’m a little peckish, a handful of cereal will usually do me. If I’m hungry, I’ll try a granola bar. If I’m getting up early for a race, I’ll usually have scrambled eggs and toast or a granola bar. I also drink water before I go out, even if early in the morning. Did a 3.35-mile run with my boyfriend today, we had to stop halfway through because he felt ill from the coffee and cereal we had for breakfast (I definitely was starting to be a little sick of the milk but was pushing through since it wasn’t so bad). I think I might need to just go out on my own for morning runs when I’m visiting him—he likes to eat and then wait until he’s digested a bit, and I’d rather just go and come back and be able to take my time getting ready for the day.

##### Conjunction Example 2

...I feel that pain man, 3 kids and a wife myself! Have made it a priority to get in my exercise, and told the family that as well. I figure a healthier Dad is better than a Fat/Dead from a Heart Attack one, LMAO! My job makes it a bit easier; I can work a couple hours into the middle of the day for exercise. If I had to wait until evening, I’m sure I wouldn’t be able to do it consistently, and I am not a morning person.

## Discussion

Overall, results of this study suggest that there was a significant impact of time on weight loss for those individuals utilizing the DropPounds forum and tracking system. Beyond that, we also found that the style of utterances within an online weight loss forum is associated with weekly fluctuations in weight. Based on the models we ran, it appears that increased use of past-tense verbs and words related to motion were associated with lower weekly weights whereas increased use of conjunctions (eg, and, but, whereas) and exclusion words (eg, but, without, exclude) were both associated with higher weight during the weeks when these utterances were used more. In general, our results have generally verified the finding that style words have much more predictive power than content words [13].

It is important to be mindful that in the current analyses time was taken into account, but the analyses are largely more akin to a repeated set of cross-sectional analyses as opposed to longitudinal analysis. This is because although weight and posts were measured frequently, the actual associations are being explored when both were measured at the same time. Although it is plausible to create models that allow for temporal relations to be determined (eg, models whereby you lag your predictor such that the previous instance of your predictor predicts the current instance of your outcome variable [35]), this dataset with its frequent missing data is not well suited for this type of lagged analysis. Because this is more akin to cross-sectional analyses, it is impossible to determine the directionality of the effects. With this in mind, the quotation examples appear to provide some insights that seem to imply the likelihood that the posts are likely more driven by the weight loss than the posts driving weight loss. This is based on the fact that all the posts, not surprisingly, have some degree of self-reflection and “sense-making” of weight loss efforts, including behaviors such as exercise.

Even if these results are more indicative of providing explanations for weight loss, they still do provide some insights on the types of activities that individuals utilizing a weight loss forum might want to participate in and be a key target to facilitate within the forums. For example, it appears that there might be some advantage with reflecting on the past to place current efforts at weight loss into perspective based on the association with past-tense verbs. Based on this, a possible future design implication to explore is to facilitate individuals in this sort of reflective “weight loss journey” process. Related to the motion words, it seems logical to conclude that running some type of race and exercise more generally is a valuable strategy to use to foster weight loss. This fits with research suggesting that diet and exercise together appear to be particularly useful for promoting weight loss [36]. From a system design standpoint, a logical strategy to incorporate into online forums could be the facilitation of individuals into partaking races and other exercise routines more actively.

Related to the constructs that were associated with weight gain (ie, exclusion and conjunctions), the previous research on these variables, particularly exclusion, tends to be associated with increased honesty. It does seem quite plausible that this is simply a reflection of “owning up” to any current weight gain. Because conducting the lagged explorations is not possible in the current dataset based on missing data, it is not possible to examine if these sorts of observations would result in better weight loss the following week. It is plausible, however, that this sort of effect would happen. Regardless, these word types (ie, exclusion and conjunctions) might be valuable identifiers of honesty within the system, which could be used to better understand the current psychological state of users of the forum.

Because this work utilizes secondary data, results suggesting that there was weight loss followed by a leveling off of weight should be considered more descriptive than causative. As already articulated, the analyses controlled for time, but the analyses are more akin to cross-sectional analyses than longitudinal analyses particularly when exploring the relation between utterances and weight. As such, directionality of the effects cannot be determined. Further, the current sample is a convenience sample of real-world users of the DropPounds system. Based on this, there is a natural selection bias in the sample. Despite these limitations, this convenience sample does provide ecological validity in that it is a real-world sample of individuals actively working on weight loss. As such, it is valuable dataset for the specific questions we sought to answer focused on understanding how natural utterances in an online forum are related to fluctuations in weight.

To the best of our knowledge, this study represents the first time styles of utterances within an online forum have been associated with fluctuations in weight. These exploratory results provide empirical evidence for possible interactions to specifically target and facilitate within online weight loss forums, such as participating in races/marathons and reflecting on current weight relative to past experience. In addition, results suggest that certain types of utterances (ie, exclusion in particular) may be a valuable proxy for identifying when a person is making an honest appraisal of their current difficulties with weight. This information could be used to help flag individuals that might need additional help within these online forums. Future work could explore trying to implicate these lessons into the design of more robust online forum interactions.

## Abbreviations

AIC: Akaike information criterion
BIC: Bayesian information criterion
LIWC: Language Inquiry and Word Count
